# The presence of personality traits of borderline personality disorder in anorexia nervosa and obesity

**DOI:** 10.1192/j.eurpsy.2024.1165

**Published:** 2024-08-27

**Authors:** F. Mustač, T. Galijašević, E. Podolski, M. Matovinović, D. Marčinko

**Affiliations:** ^1^Department of Psychiatry and Psychological Medicine, University Hospital Centre Zagreb; ^2^School of Medicine; ^3^Department Of Endocrinology and Diabetology, Internal Clinic, University Hospital Centre Zagreb, Zagreb, Croatia

## Abstract

**Introduction:**

While in eating disorders such as anorexia nervosa, the comorbidity of pathological personality traits is very common, and accordingly the personality traits of borderline personality disorder is considered very frequent and coexisting. The treatment of anorexia nervosa is based primarily on the psychotherapy and work on pathological personality traits even more than the treatment of the syndrome and the consequences of malnutrition itself. That achieves a longer-term and more reliable solution than symptomatic treatment of anorexia nervosa which usually does not bring satisfactory results. On the other hand, in patients with obesity, pathological personality traits, especially those of borderline personality disorder, are still very rarely associated, since obesity is usually not even considered a disorder, but a variation in the population.

**Objectives:**

The aim of this paper is to investigate the pathological personality traits of borderline personality disorder in people with obesity.

**Methods:**

Investigating relevant scientific and professional literature from the field of personality pathology and eating disorders.

**Results:**

When obesity is related to impulse control disorder in the sense of emotional eating under increased stress according to today’s relevant literature, it can definitely be related to personality traits of borderline personality disorder, i.e. the presence of elements of borderline personality organization and prementalization models. Such an inability to deal with negative emotions such as increased anxiety or rejection sensitivity, which results in overeating and the related feeling of shame that overwhelms the person, regardless of whether he/she/they has any of the certain forms of compulsive behaviour afterwards, can be related to impulsive behaviour and the “all or nothing” way of thoughts. This is also confirmed by cases when certain people have a history of both one and the other disorder. Thus, some people have, for example, malnutrition in adolescence as part of anorexia nervosa, only to have problems with obesity after some time with a healthy body mass.

**Conclusions:**

Since pathological personality traits in people with anorexia nervosa and obesity give indications of common characteristics in the form of borderline personality disorder traits, i.e. borderline personality organization and prementalization models in both disorders, future research will certainly shed light on the connection between these eating disorders.

**Disclosure of Interest:**

None Declared

